# Bringing stakeholders together for urban health equity: hallmarks of a compromised process

**DOI:** 10.1186/s12939-015-0252-1

**Published:** 2015-11-20

**Authors:** Amy S. Katz, Rebecca M. Cheff, Patricia O’Campo

**Affiliations:** Centre for Research on Inner City Health, St. Michael’s Hospital, 30 Bond Street, M5B 1W8 Toronto, ON Canada

**Keywords:** Health equity, Participatory processes, Community engagement, Stakeholder engagement, Urban health equity, Urban governance, Neoliberalism and cities

## Abstract

There is a global trend towards the use of ad hoc participation processes that seek to engage grassroots stakeholders in decisions related to municipal infrastructure, land use and services. We present the results of a scholarly literature review examining 14 articles detailing specific cases of these processes to contribute to the discussion regarding their utility in advancing health equity. We explore hallmarks of compromised processes, potential harms to grassroots stakeholders, and potential mitigating factors. We conclude that participation processes often cut off participation following the planning phase at the point of implementation, limiting convener accountability to grassroots stakeholders, and, further, that where participation processes yield gains, these are often due to independent grassroots action. Given the emphasis on participation in health equity discourse, this study seeks to provide a real world exploration of the pitfalls and potential harms of participation processes that is relevant to health equity theory and practice.

## Background

In most cities, some people have more access than others to elements that are fundamental to individual and community health. When people and communities do not have access to what they need to thrive, it is not by choice, but rather that they are prevented from exercising meaningful input into decisions affecting the distribution of resources and shape of the society [1–4].

Currently, there is a trend towards the use of ad hoc participation processes^1^ in relationship to municipal infrastructure, land use and services [5, 6]. These processes are often characterized as the expression of a shift from local government to local governance [7], whereby a range of stakeholders such as private businesses, non-profits, community residents and the state are charged with working together to plan and/or implement various projects.

Some have posited that these and related participation processes can help to address urban health inequities by allowing what are variously termed marginalized, equity-seeking, vulnerable or disadvantaged groups (or, simply, 'communities') to have more of a say in urban governance, thereby leading to more equitable access to the social determinants of health [1].

Others argue that the emerging crop of government-convened, ad hoc participation processes generally have little potential to shift power relations [5, 8]. This serves to challenge their potential to address health inequities within cities, as evidence demonstrates that power relations enforced through overlapping structures such as (but not limited to): colonization [9]; racism [9–11]; misogyny; constructions of citizenship [11, 12] and economic injustice and exploitation [1, 2, 11] are responsible for many aspects of preventable ill health.

In this paper, we hope to contribute to the discussion regarding the utility of participation processes in advancing urban health equity by closely reading the processes themselves. To do this, we present the results of a scholarly literature review examining cases of ad hoc participation processes convened by governments and with some relationship to social determinants of health (e.g. affordable housing, neighbourhood infrastructure, etc.), captured in articles examining processes in cities in Australia (1), Austria (1), Costa Rica (1), Denmark (1), India (1), Italy (1), South Africa (1), UK (1) and the US (6). In doing so, we uncover hallmarks of processes with compromised potential to deliver equitable decision-making^2^ and concrete outcomes^3^ and identify potential harms inflicted on grassroots stakeholders^4^ by the process of participation itself.

This paper focuses on ad hoc participation processes such as consultations, advisory groups and multi-stage, often multi-year planning processes, principally related to neighbourhood planning and/or place-based strategies and without the binding power of legislative deliberation. It does not examine institutionalized grassroots participation in municipal government such as participatory budgeting [13–15] or the formal inclusion of community organizations in binding decision-making processes [16].

## Methods

We conducted a search of all English articles published between 1990 and June 2015 in the Web of Science (Thomson Reuters) database using the following topic words (i.e. title, abstract, author keywords, and Keywords Plus®): (urban OR city OR cities or metropolitan) AND ((grassroots or stakeholder*) NEAR (collaborat* or consult* or partner* or engage* or participat* or “work* together” or equit* or inequit* or barrier*)). 605 articles were identified through this initial search.

We then reviewed article abstracts, including articles published largely from 2005 to June 2015 that: focused on specific cases of government-convened participation processes in cities; were expected to deliver outcomes within the description period; and, that had some relationship to social determinants of health. We focused on those cases that looked at processes with broad implications, eliminating those concerned with discrete projects (eg. community gardens).

To focus on the trend towards neighbourhood planning, we eliminated cases concerned with climate change planning or mitigation, disaster relief or response, education, tourism and specific health outcomes (eg. smoking cessation). An additional 4 articles were identified through reference lists and additional searches. These combined search strategies resulted in 14 articles that fit the study inclusion criteria. We coded the articles included according to themes suggested in the broader literature, and themes that emerged in and across the papers themselves, in order to identify some hallmarks of problematic processes and explore potential harms.

We wish to emphasize that our findings will only have useful local application in relationship to health equity when considered through the filter of local conditions, as inequities and their historical creation and contemporary maintenance will be, at least in part, local in nature, as will modes of resistance. Georgina Blakely writes, “We need to acknowledge… how similar global trends are experienced and embedded in each locality in different ways, while recognizing some common features are evident” ([17], p. 133). Our goal is to draw out these common features while underlining the critical role of local context in any interpretation of these findings.

## Review

In this section, we elaborate on key issues extracted from the 14 cases included in our study. We summarize these issues, supplemented with issues raised in the broader literature, in two tables. Table 1 represents factors that impede the ability of participation processes to generate concrete outcomes informed by equitable decision-making. Table 2 examines potential harms generated by the process of participation itself. As our goal was to gage these processes in terms of their potential to improve health equity, we consider a ‘compromised process’ one which fails to deliver substantially on the goals of grassroots stakeholders. Both tables include specific lessons drawn directly from cases reviewed, data from the broader literature, and aggregate observations based on both the cases and the broader literature.

**Table 1:** Barriers to equitable decision-making and concrete health equity outcomes in the context of participation processes

Cluster A: Process structure, resourcing and capacity	Cluster B: Stakeholder representation and participation
o Process is not binding [23–26]. o Process is occurring at the wrong scale (e.g. relevant decisions/resources concentrated at another level of government or inequities generated by globalized processes) [5, 23, 28]. o Process is entirely controlled by convener [23]. o Limited range of potential outcomes (due to convener or funder restrictions, pre-existing policy decisions, framing information provided to participants, etc.) [5, 21, 22, 24]. o Key conversations had/decisions made outside of process [24]. o Not enough time for discussion, reflection; process too short and/or insufficiently iterative [23, 32]. o No or insufficient resources provided to ensure bargaining power of grassroots stakeholders (e.g. outreach, facilitation, community organizing, training) [23]. o No or insufficient resources for neighbourhood-level data collection (e.g. population and infrastructure) [26]. o Inability to deliver targeted outcomes (e.g. lack of capacity to address land title, hire independent consultants) [18, 23].	o Diversity of grassroots stakeholders not represented, including diversity *within* distinct groups [24]. o Grassroots stakeholders represented by groups funded by government or industry [5, 18, 19]. o Process favours stakeholders with pre-exiting capacity to organize/participate [22–24]. o Bureaucracy, planning professionals and/or industry over-represented in numbers or voice [22, 24]. o Overall group membership reproduces existing power structures [20, 21]. o Lack of practical resources such as child care, meals and transportation limits participation by grassroots stakeholders [39]. o Forum time/location limits participation by grassroots stakeholders [18]. o Demands for volunteer labour place a particular burden on or serve to limit participation of those with little to no leisure time [20, 22, 24].
Cluster C: Information-sharing and gathering	Cluster D: Implementation
o Infrequent information sharing (gaps and silences) [26]. o Information shared is vague (e.g. language is confusing or inconsistent, lack of detail) [26]. o Information and meetings in language of dominant group [18, 20]. o Information sharing limits audience (e.g. only sent to people via email) [18, 25]. o Technical information provided by consultants who lack independence (e.g. funded by industry) [18]. o Forums structured so as to limit types of input and/or speakers considered legitimate [22, 24]. o Forms/paperwork built into process limit types of input [32]. o Excessive focus on consensus, conflict seen as antithetical to process [21, 24].	o Participatory aspect of process includes planning but not implementation [19, 23, 26]. o No plan to monitor and report back on implementation. o No accountability to stakeholders following deliberation process.

Grassroots stakeholders do not have sufficient independence and/or capacity to apply pressure to process/organize outside of process

**Table 2:** Potential harms of participation

Cluster A: Delegated control	Cluster B: Demobilization
o Behaviour of grassroots stakeholders shaped by process, thereby containing dissent (e.g. grassroots stakeholders positioned to act as ‘gatekeepers’ rather than as community representatives; meeting norms discourage certain types of input; paperwork/reporting begins to shape thinking) [5, 19, 22]. o Energy of grassroots groups occupied by process, thereby containing dissent. o Process embeds and transmits logic of status quo (e.g. neoliberalism, colonialism, structural racism and misogyny, etc.).	o Participants experience frustration and/or burn-out. [23, 25, 26]. o Participants lose faith in participatory processes/participation [22]. o Community leaders who have championed process lose credibility [23]. o Energy of grassroots groups sapped by process, thereby diminishing capacity. o Participants/groups face sanctions for resisting process as defined (see below).
Cluster C: Contraction of state role in public service delivery/regulation	Cluster D: Sanctions
o Success of project relies on free labour of participants. o Project fails to address and/or distracts from broader, systemic processes due to focus on ‘micropolitics’ [5, 21].	The broader literature emphasizes that participants resisting or building alternatives to official processes can, in some contexts, face sanctions ranging from loss of paid work [19] to imprisonment and violence [27].

### Grassroots stakeholder representation

The cases reviewed revealed common issues related to stakeholder representation in participation processes. Community organizations (COs) or Non-Governmental Organizations (NGOs) were often invited to serve as proxies for some constituencies and/or interests. In some cases, funding from industry [18] or government [5, 19] had the potential to make it difficult for COs/NGOs to engage in strong, independent advocacy.

In addition, despite explicit or implicit equity goals, it was often unclear as to how equity played out on the level of stakeholder representation. Most papers did not provide detail in terms of how power relations impacted representation (and, in some cases, it seemed disaggregated data was not collected by the City). A minority of cases, however, explicitly indicated that resident involvement was largely middle class [20, 21], with one indicating that participation processes served to actively exclude people with less socio-economic status [20].

Other issues included overrepresentation of people with pre-existing capacity to organize [22–24], and underrepresentation of the diversity within communities, with individuals asked to speak for broad polities without the time and resources to gather input [24].

In at least one case, despite outreach efforts, grassroots stakeholders did not engage widely with the process, which largely attracted planning professionals. At the same time, the government used the ‘representativeness’ of the process (which they did not track) to demonstrate that they had a broad mandate for their planning strategy [24].

### Grassroots stakeholder participation

Once the stakeholder tables were assembled, additional obstacles emerged for grassroots stakeholders. Some came down to meeting arrangements, for example: meetings scheduled while people were working [18]; information communicated in a way that restricted potential recipients [18, 25]; meetings conducted in the language of the dominant group and/or elites [18, 20]; and, demands for volunteer labour which placed a particular burden on or served to exclude those with little to no leisure time [20, 22, 24].

Many cases pointed to subtle strands of control woven throughout processes with the potential to shape behaviour and/or input.^5^ Some indicated a pervasive emphasis on consensus, one with the effect of discouraging dissent [21, 24] and favouring the views of more powerful actors [22, 24]. Others demonstrated that grassroots stakeholders can be placed in the role of ‘gatekeepers,’ sandwiched between representing their community and representing the process itself [19], once again potentially dampening dissent.

Paperwork was also mentioned as a mode of control by which, “auditing requirements imposed from outside were gradually internalized by communities themselves…” [5], p. 304. In addition, some authors gestured to the ways in which meeting structure attempted to shape an ideal, normative participant – someone who engages ‘well’ with institutional processes and only suggests ideas that are ‘reasonable’:

Power relations are expressed through, for example, what counts as valid knowledge, and thereby has an effect on what is perceived as “truth” or “the reality”. In this way, different forms of communication and knowledge such as emotional expressions or storytelling are often marginalized… [22], p. 1089

These various strands of control can have a concrete impact on outcomes, as in one case where, “Discursive exclusion exterminated points of view that were deemed unrealistic, unobtainable, politically controversial or otherwise non-desirable by the authorities” [22], p. 1094.

Several cases described negative consequences for both grassroots stakeholders and community capacity more broadly. These included the discrediting of community leaders who initially championed a process-gone-wrong [23], and burn out, frustration and rage, in particular when time-consuming processes proved to be opaque and/or relatively unproductive [23, 25, 26]:

High officials with open arms and a smile on their face welcomed us and told us that despite the justified delays, we would forge ahead. It was all a cruel mockery of our trust, time and dedication. [23], p. 74

One case alluded to the possibility that grassroots stakeholders will experience participation processes in the end as a wearying reminder of their lack of formal political power, potentially foreclosing future participation:

They found it a banal confirmation of their fear that at the end of the day decisions would after all be made at the town hall and not in the neighbourhood as promised. [22], p. 1095

It should also be noted that one case pointed to the risk of losing paid work when choosing to challenge official processes and decisions [19], while the broader literature indicates, depending on the context, the potential for severe and ongoing sanctions such as imprisonment and violence [27].

### Process

#### Political will and technical problems

Most processes failed to some degree to deliver on the promise of participation from the point of view of grassroots stakeholders, often due to the fact that they were not binding. In some cases, multi-year planning exercises were discarded in whole or in part following a change in government, or key planned outcomes failed to materialize, without clear consequences for conveners [23–26].

While lack of political will was a frequent reason for failure to implement a given planning process, some governments and organizations seemed to lack the technical capacity to deliver outcomes, for example, the ability to address the question of land title [23] or the resources to hire consultants independent of industry [18].

It was difficult in some cases, however, to distinguish between a technical problem and a failure of political will. In one case, for example, it became impossible to track the implementation of a neighbourhood revitalization plan in part due to the fact that the local government seemed not to have adequately mapped and classified the properties at the centre of the process. Grassroots stakeholders and academics, however, set out to prove that this was not, in fact, a technical obstacle. The group found a way to map and classify these properties themselves, raising the question of:

…why so much time, money, and effort was invested into creating a consensus-building planning process and so little time, money and effort was invested into monitoring the implementation of the consensus-built plan. [26], p. 190

As mentioned above, some planning processes provided for little investment in participation at the implementation stage [19, 23, 26], potentially impacting the ability of grassroots stakeholders to hold conveners to account. While this may present as a technical problem (a badly designed process), the frequency with which it occurred raises the question of whether this issue, as well, is related to political will.

#### Dissonances: scale and tools

Some cases highlighted inter-related problems of scale. For example, participation processes occurring at the local scale can be charged with addressing issues for which responsibility and/or resources principally rest with other orders of government [5, 23], or compensating for ‘declining resources allocated from higher tiers of the state’ [28], p. 125.

Some cases highlighted the problem of scale at the level of impact, arguing that participation processes only offer the opportunity to shape ‘micropolitics’ [5, 21] while broader issues with the potential to make a transformative change are left off the table:

The trouble is when you organize and try and come up with a solution, the solution isn’t what the people who are in power are saying they want to hear, like the problem of affordable housing […] and the price of land, who gets it and what is built on it – these big issues never come up at community forum meetings. [5], p. 304

Finally, at least one case identified a disjuncture between problems and solutions whereby a participant saw social inclusion posited as a solution to the problem of public sector contraction:

I think to say that a community that isn’t successful suffers from a lack of social capital is blaming the victim, saying that you’re not talking to each other enough, you’re not getting involved enough – well, frankly, you shouldn’t be closing the local hospital, pal. [5], p. 305

### Independent grassroots action

The ability of grassroots stakeholders to exit the official process and apply independent pressure was integral to the achievement of concrete gains. In case after case, grassroots stakeholders chose to go outside the confines of an official participation process in order to meet their goals [18, 19, 21, 23, 25, 26]:

Indeed, much of the more fruitful negotiations in terms of achieving real outcomes took place outside the pre-designed formal procedure, normally when community leaders took the initiative and used protest measures, or threatened to use them. [23], p.76

Sometimes, this pressure was facilitated, in an indirect way, by the participation process itself, as grassroots groups built and/or maintained through associated funding pushed back against decisions [19] or went off plan [28] to achieve their goals. Far more often, pressure was facilitated by what seemed to be pre-existing community organization [18, 23, 25].

Even where there was no or little direct conflict, processes that were characterized as somewhat or very successful *on their own terms* were defined by an infusion of dollars invested over the long-term in what seemed to be relatively independent community organization [28, 29].

## Discussion

Two clear findings came out from the cases reviewed. The first: participatory planning processes often cut off participation (or even activity) at the implementation phase, making it difficult for grassroots stakeholders to hold conveners to account. The second: where participation processes yielded gains from the perspective of grassroots stakeholders, these were often due to independent grassroots action. These two findings are related, and reveal a tension we would argue underlies our findings, that of power relations.

### The role of opacity

The sharing – or non-sharing – of different types of information is one way to map the play of power relations in the context of participation processes. For example, while grassroots stakeholder goals might be relatively legible, like the demand for housing [23, 26], institutional goals are not always clearly articulated. Instead, institutional goals are often expressed through action at different points – failure to implement a planning process in whole or in part [23–26], the ‘writing out’ of specific grassroots stakeholder demands [22], the defunding of dissenting organizations [19]. Grassroots stakeholders, therefore, generally find out what’s going to happen *after it happens*, or *when they make it happen* through pushing back [19, 21, 23, 25].

Some scholars suggest that partnership-based initiatives such as ad hoc participation processes can be interpreted as an emerging form of loosely-defined, democratically unaccountable decision-making, characterized as a shift from government to governance [5, 7, 8, 21, 30, 31]. These new forms of urban governance can attempt to compensate for some of the ‘accumulating economic and social tensions’ produced by neoliberal economic practices ([30], p. 4). At the same time, they can be so marinated in the logic of neoliberalism and other dominant logics that they exclude – without explicitly saying they are doing so – solutions that would serve to transform interrelated economic and social relations.

Adding to the confusion, ad hoc participation processes and other partnership-based initiatives seem to have dual functions. On one hand, they attempt to mitigate the consequences of neoliberal economic practices (while severely circumscribing the scope of this mitigation). On the other hand, the literature makes clear that new forms of urban governance are strongly linked to the roll-out of neoliberal economic practices such as the contraction of public sector services [7], an emphasis on entrepreneurialism [28] and a focus on volunteer labour [5]. These dual functions – mitigation and roll-out – are, however, likely rarely stated. As a result, grassroots stakeholders are compelled to decipher institutional responses not in dialogue but in action: once the facts on the ground have already been established.

Some ad hoc participation processes and their attendant vocabularies, then, can be read, in part, as the expression of attempts to both enact and mitigate neoliberal economic practices while simultaneously avoiding the explicit articulation of these goals. The result is a riot of dissonances, loose strings, glosses and misdirects. Precise indicators established during a planning process are replaced with vague catch-alls during the implementation phase [26]. Undefined notions of ‘inclusion’ and ‘social capital’ are positioned as having the capacity to take on social and economic issues [5] produced by overlapping power structures such as neoliberalism and colonialism. Elaborate planning processes peter off into silence.

#### The role of independent action

Some critical scholars suggest there is little point in engaging if the local government has a neoliberal orientation [8], while others find opportunity even in compromised and/or clearly neoliberal processes [28, 32]. Almost all, however, emphasize the importance of the independence and capacity of grassroots stakeholders. Sherry Arnstein, writing in 1969, points out that grassroots stakeholders require both independence and access to resources like lawyers, community workers, etc. in order to play a genuine role in participation processes [3]. Marcelo Lopes de Souza suggests that the presence of both autonomous civil society groups and genuinely non-conservative parties is conducive to the possibility of productive intersections between grassroots stakeholders and city processes [33].

There is some evidence, as well, that grassroots stakeholders who maintain independence can see collateral benefits from participation processes, for example, through using them as: opportunities to learn more about how government/governance works [33]; sites at which to meet other grassroots stakeholders in order to build independent organization efforts; sites at which to build solidarity in opposition to a common challenge [23]; and/or opportunities to impact policy by shifting attitudes of elected officials and bureaucrats [11, 32].

The importance of independent grassroots groups is evidenced more generally in relationship to issues of health equity. Writing about not-for-profits in the US, contributors to the anthology *The Revolution Will Not Be Funded: Beyond the Non-Profit Industrial Complex* correlate the autonomy of movements and groups directly to effectiveness. In ‘Native Organizing Before the Non-Profit Industrial Complex,’ Madonna Thunder Hawk draws a clear link between independent organizing and the ability to take on environmental racism and related negative health outcomes (for example, due to contaminated water) [34]. In relationship to housing policy in South Africa, Richard Pithouse suggests that:

…it is in the struggles of progressive poor people’s organizations to increase their power and to reduce that of elites across the triad of state, capital and civil society that academic urbanists will have the best prospects for effecting positive change… [35], p12

In the context of participation processes in cities, we conclude that, if effective intersections are to occur, it will be due to independent grassroots organizing and grassroots urban planning that occurs, as Souza puts it, “…sometimes *together with* the local state apparatus, sometimes *despite* the state, sometimes *against* the state…” [33].

### Limitations

While a minority of cases delivered what the literature characterized as substantially positive outcomes, our goal was to draw out process patterns that serve to compromise success from the point of view of grassroots stakeholders. In addition, it is possible that the literature itself is more focused on cases that go wrong than cases that go right. As a result, the paper might serve to under-emphasize the positive outcomes that can result from some of these processes. As one example, one case [23] alludes to parallel processes in rural areas that saw more success.

At the same time, it is certain that this paper dramatically under-represents potential harms to those actively pushing back against state-sponsored processes or decisions. Incidences of severe harm – from professional sanction to imprisonment and death – were not largely present (or, at least, documented) in cases described in the slice of peer-reviewed literature that fit into our inclusion criteria. The broader academic literature, social media and the daily news all make clear, however, that communities and individuals face a range of harsh sanctions for registering dissent, and local context is key to interpreting this potential.

We also wish to point out that we did not seek to corroborate or contradict the authors’ characterizations of the cases. It is for this reason, in part, that we do not explore cases in depth in the paper, or name cities. Our goal was to extract patterns from the literature as opposed to undertake a deep exploration of individual processes.

The literature also reflects power structures in terms of who is able to enter academia, and academic structures and strictures in general. As we have not delved deeply into the grey literature, we cannot contend that we have provided a complete portrait of the pitfalls of these processes. Rather, we have sought to characterize *what is in a slice of the peer*-*reviewed literature* to fill in one piece of a vastly richer puzzle.

Finally, we wish to reiterate that this review includes 14 studies from nine countries, six of which are members of the Organisation for Economic Co-operation and Development (OECD). As a result, the studies reviewed do not offer a universal snapshot of urban participation processes and our findings cannot be meaningfully interpreted outside of particular contexts. As one example, in Toronto, where we live and work, efforts to define and push for ‘equitable urban decision-making’ must take into account historical and current impacts of colonialism, and the rights of heterogeneous Indigenous communities and nations to self-determination [36, 37]. Our hope is that these patterns, denuded of context, will come to life where relevant when re-embedded through local interpretation.

### Strengths

This paper represents a novel attempt to marry a health equity framework to urban planning, geography and participation literature. Given the emphasis on participation in health equity discourse, an exploration of the pitfalls and potential harms of these processes is essential to health equity theory and process.

## Conclusion

It stands to reason that any process designed to address urban health inequities would take up the interrelated dynamics that generate inequities in the first place such as colonialism, racism, misogyny, restricted constructions of citizenship and economic injustice. But the question of how to take up these dynamics is not, fundamentally, a technical one. Rather, we contend that the problems manifest in participation processes are largely (although not exclusively) expressions of underlying political realities, telegraphing embedded truths rather than potential technical fixes. Richard Pithouse writes about ‘progressive polic[ies] without progressive politics’ [35]. We wish to draw a link between his observation and the phenomenon of collaborative processes without progressive politics and therefore collaborative outcomes:

…the newly appointed City manager […] acknowledged that the end result of the plan may not have been part of the original vision and that “the laundry list of projects is not as important as the process by which we undertook to get things done…” [26], p.187

This fascinating statement encapsulates, to some degree, the spirit of a compromised participation process: it was not necessarily meant to accomplish the outcomes discussed. It stands in the service of opaque goals, a performance that likely confuses even some of those recommending or producing it [30, 38]. As a result, our hope with this work is to make some contribution to the legibility of ad hoc, urban participation processes in neoliberal contexts.

## Footnotes

1 For the purposes of the paper, we define a ‘participation process’ as one convened by a government with the stated goal of including grassroots stakeholders in the process itself. While we call these participation processes, they have been variously labelled with terms like collaboration, partnership, community engagement, stakeholder engagement, and popular participation. We choose the noun 'participation' as opposed to the adjective 'participatory' to suggest that the stated intent of creating a participatory process does not always result in genuine participation.

2 We consider an equitable decision-making process one that is transparent, and that does not replicate unequal power relations either through stakeholder representation or the ways in which stakeholder voices/opinions are filtered through the process and impact the final outcome.

3 A concrete outcome can be considered a traceable result of a participation process, in keeping with both the scope and spirit of its stated mandate. For the purposes of this paper this was most often applied to the distribution of resources including the development of infrastructure. It could also be seen to include the change or development of regulations, practices and/or policies that are subsequently enforced.

4 While processes with the stated aim of increasing participation vary widely in terms of scope and goals, many articulate a desire to include grassroots participation in the process itself. For the purpose of this paper, we define a grassroots stakeholder as an individual, group or organization that is not entrenched on balance in current formal or informal power structures in relationship to the issue at hand. While grassroots stakeholders may include aspects of 'civil society,' it should be noted that in many cases NGOs cannot be considered proxies for communities. In addition, not all grassroots groups are emancipatory and/or progressive in nature (i.e. there can be reactionary grassroots groups and movements) [40, 41].

5 Maureen Taylor (2007) offers a precis of ‘governmentality’ ([5], p. 300), a concept which attempts to theorize the ways in which state control can be enacted at arm’s length and through an intricate series of practices, including, in some cases, participation processes. Renuka Sooknanan (2000) offers a case study of governmentality in action related to, “…the ways in which state funding and community-state partnerships regulate immigrant women’s communities” [42]. See also Swyngedouw (2005) [31] and Blakeley (2010) [17] for more on governmentality.
